# A multiplex Mtb-specific FluoroSpot assay measuring IFNγ, IL-2, and TNF-secreting cells can improve accuracy and differentiation across the tuberculosis spectrum

**DOI:** 10.1128/jcm.00894-25

**Published:** 2025-10-29

**Authors:** Elin Folkesson, Fariba Foroogh, Linn Kleberg, Vera Kjellgren, Mathilda Jakobsson, Lisann Grunewald, Jens Hellberg, Jennifer Ryberg, Zahra Maher, Carolina Sousa Silva, Maia S. Gower, Hans Grönlund, Margarida Correia-Neves, Bartek Makower, Gunilla Källenius, Judith Bruchfeld, Christopher Sundling

**Affiliations:** 1 Division of Infectious Diseases, Department of Medicine Solna, Karolinska Institutet 27106, Stockholm, Sweden; 2 Department of Infectious Diseases, Karolinska University Hospital 59562, Stockholm, Sweden; 3 Center for Molecular Medicine, Karolinska University Hospital 59562, Stockholm, Sweden; 4 Therapeutic Immune Design, Department of Clinical Neuroscience, Karolinska Institutet 27106, Stockholm, Sweden; 5 Life and Health Sciences Research Institute (ICVS), School of Medicine, University of Minho 224744, Braga, Portugal; 6 ICVS/3B’s-PT Government Associate Laboratory, Braga/Guimarães, Portugal; 7 Mabtech AB 650213 , Nacka Strand, Sweden; The University of North Carolina at Chapel Hill School of Medicine, Chapel Hill, North Carolina, USA

**Keywords:** tuberculosis, mycobacterium, diagnostics, T cells, cytokines, immuno-assay, FluoroSpot

## Abstract

Tuberculosis (TB) remains a major global health challenge, with current diagnostic tools unable to reliably distinguish between individuals at different stages of the TB spectrum of infection (TBI) and disease (TBD). Interferon-gamma release assays (IGRAs), while commonly used, are limited by their reliance on a single cytokine and inability to differentiate disease states. In this study, we evaluated a multiplex FluoroSpot assay that simultaneously detects interferon gamma (IFNγ), interleukin (IL)2, and tumor necrosis factor (TNF)-secretion in response to *Mycobacterium tuberculosis* (*Mtb*) antigens ESAT-6, CFP-10, and EspC. PBMCs from individuals with TBD (*n* = 24), TBI (*n* = 64), and IGRA-negative controls (*n* = 26) were analyzed. The assay demonstrated high sensitivity and specificity, particularly through the detection of triple cytokine-secreting T cells (IFNγ/IL-2/TNF), minimizing false-positive rates. Moreover, distinct cytokine secretion patterns were associated with different stages of infection. Individuals with TBD had more IFNγ/TNF-producing cells, while those with TBI had more cells producing IFNγ/IL-2 or IL-2/TNF. Separating individuals with TBI based on time since likely Mtb exposure revealed that more recent infection was associated with reduced IFNγ responses and a higher proportion of IL-2/TNF-secreting cells. The use of spot volume analysis provided semi-quantitative insights into cytokine production. These findings suggest that multiplex cytokine profiling can improve TB diagnostics and offer a deeper understanding of host immune responses across the TB spectrum. Including non-IFNγ responses may be particularly valuable in identifying individuals at higher risk of disease progression, such as recently infected individuals, and in populations where current diagnostics underperform.

**IMPORTANCE:**

Accurate diagnosis of *Mycobacterium tuberculosis* (*Mtb*) infection remains a cornerstone in tuberculosis (TB) control. Current interferon-gamma release assays (IGRAs) lack the ability to distinguish between individuals at different stages of the TB infection spectrum, limiting their utility. This study evaluates a multiplex FluoroSpot assay that simultaneously detects interferon gamma (IFNγ), interleukin 2, and tumor necrosis factor secretion in response to *Mtb*-specific antigens ESAT-6, CFP-10, and EspC. The assay demonstrated improved performance compared to standard IGRA methods, particularly through the identification of triple cytokine-secreting T cells. Importantly, it revealed distinct cytokine profiles associated with different stages of TB infection, offering potential for improved risk stratification and infection monitoring. These findings support the FluoroSpot assay as a promising tool for enhancing TB diagnostics and understanding host immune responses. Its application could be especially valuable in contact tracing, where *Mtb*-specific T-cell responses may not yet produce detectable amounts of IFNγ.

## INTRODUCTION

Tuberculosis (TB) remains a significant public health threat with an estimated 10.8 million cases of TB disease (TBD) in 2023, resulting in 1.25 million deaths, thus making it the leading cause of mortality caused by a single infectious agent (1). The persistently high incidence is the result of ongoing transmission, but also due to the ability of *Mycobacterium tuberculosis* (*Mtb*) to establish a TB infection (TBI) that will most often pass unnoticed but after a variable length of time can resurge and cause TBD. Current estimations infer that approximately 25% of the world’s population has TBI, defined as reactivity for Mtb in immunological tests without clinical or radiological evidence of TBD (2). This reservoir of possible future incident TB cases needs to be addressed to achieve the end TB 2035 goals (3), aiming to reduce TB incidence by 90% and TB mortality by 95% compared to 2015 numbers (4).

A historic simplified notion of *Mtb* as either causing TBD (symptomatic disease) or TBI (persistent non-symptomatic infection) has been replaced with the understanding that these are different states of a disease spectrum (5–7). The balance between *Mtb*-specific immunological control and mycobacterial survival determines the outcome in each patient, with around 5% of *Mtb*-infected individuals progressing to TBD within the first couple of years after infection and an estimated 10% lifetime risk for TBD overall (5, 7). It is not known which proportion of individuals eventually clears the infection over time, though some argue that lifelong infection is less common than previously believed (8), and currently, there are no methods to detect true persistence of viable *Mtb* (9). Instead, the diagnosis of TBI is based on indirect immunological methods, detecting a specific T-cell-mediated immune response to *Mtb* antigens, through Tuberculin Skin Tests (TST) or interferon-gamma release assays (IGRA) in blood (10). The WHO endorsed (10) IGRA tests use *Mtb* antigens ESAT-6 and CFP-10 to stimulate T cells in whole blood (QTF-Plus and WANTAI TB IGRA) or isolated peripheral blood mononuclear cells (PBMCs) (T-SPOT.*TB*), followed by interferon-γ (IFNγ) detection after 24 hours of incubation by ELISA or ELISpot, respectively. Although the IGRAs have an advantage over TST in being more specific (11), neither can differentiate between TBD and TBI or a potentially cleared infection, nor can they predict disease progression (12, 13).

In non-immunosuppressed adults, the estimated sensitivity for TBI is around 80% for TST and QFT-GIT and 90% for T-SPOT.*TB*, with lower sensitivity in immunosuppression and children (14–17), although a reference method is not available for direct comparison (14, 15). Furthermore, the positive predictive value for developing TBD is low for all tests, with T-SPOT.*TB* reaching at most 4.2% (16–18). Testing for TBI and providing preventive TB treatment (PTT) is recommended by WHO to persons living with HIV (19), household contacts of individuals with sputum positive pulmonary TB, and in anticipation of certain immunosuppressive treatments (20). To scale up PTT, more accurate testing for TBI is warranted, including tests that can differentiate recent and remote TBI and identify individuals with high risk of TB disease progression (12, 21, 22), as well as tests with high sensitivity in immunosuppressed individuals (23). Possible ways to improve current immune assays for TB could be adding other *Mtb* antigens to elicit stronger or differential responses according to *Mtb* infection stage, or measuring combinations of cytokines (24, 25). EspC, a protein involved in the secretion of *Mtb* virulence factors, elicits a strong immune response (26) and was shown to improve sensitivity for TBD when added to ESAT-6 and CFP-10 (27).

There is also increasing support for the relevance of non-IFNγ *Mtb*-specific immune responses, with the cytokines tumor necrosis factor (TNF), interleukin (IL) 2, and IFNγ-inducible protein 10 (IP-10) among the most widely investigated. For example, secretion of TNF and IL-2 in response to *Mtb*-specific (ESAT-6 and CFP-10) stimulation in extensively *Mtb*-exposed IGRA-negative individuals has been described, possibly indicating a protective TB immune response (28, 29). A recent study found the sensitivity of an assay using IP-10 and IL-2 following ESAT-6/CFP-10 stimulation equal to IFNγ in detecting TBI (21, 30, 31).

Identifying polyfunctional T cells with distinct cytokine patterns may improve diagnostic sensitivity and, in addition, potentially inform about different *Mtb* infection stages. A promising approach is the FluoroSpot technology, which builds on the ELISpot method used in T-SPOT.*TB* but uses fluorescence-labeled cytokine-specific antibodies to simultaneously detect multiple cytokines (32). In our laboratory, we have previously developed a multiplex FluoroSpot that could simultaneously assess four separate B-cell effector responses at a single-cell level (33–35). Building on this experience, we are now, in collaboration with the Swedish biotech company Mabtech AB, optimizing an *Mtb*-specific FluoroSpot (Mabtech) assay that can also detect up to four cytokines per cell. In addition to enumerating antigen-specific cells, it offers a semi-quantitative measure of the secreted cytokines by evaluating the volume of the spots (30).

In this FluoroSpot assay, we used the *Mtb* antigens ESAT-6, CFP-10, and EspC for PBMC stimulation and the cytokine panel IFNγ/IL-2/TNF to evaluate memory T-cell responses. We hypothesize that the multiplex cytokine detection would allow for increased sensitivity with the potential to differentiate disease states and T-cell function better than IFNγ alone. The main aim of this study was to evaluate this FluoroSpot method with regard to its sensitivity and specificity in TBD and TBI, and whether different stages of *Mtb* infection may be identified by patterns observed in terms of frequency, magnitude, and combination of single/double/triple cytokine-producing cells.

## RESULTS

### Study participants

A total of 114 study participants were recruited for the study, falling into three distinct groups: individuals with microbiologically verified TBD (*n* = 24), TBI (*n* = 64), and non-TB IGRA-negative controls (*n* = 26). The TBD group consisted of individuals with pulmonary TB (*n* = 13) and extrapulmonary TB (*n* = 11), with culture verification in 23 (96%) individuals in total. The study groups had similar gender distribution, age, comorbidities, and immunosuppression (Table 1). Most individuals in both TB groups were non-Swedish born, a majority from TB high-endemic countries (>100/100,000 [31]), while fewer (37%) IGRA-negative controls were from TB high-endemic countries. Individuals with TBD had significantly higher C-reactive protein (CRP) and erythrocyte sedimentation rate (ESR), and lower hemoglobin and albumin levels compared to those with TBI (Table 1).

**TABLE 1:** Clinical characteristics of the patient cohort tested with the FluoroSpot assay^*a*^

		TB disease^*d*^ (*n* = 24)	TB infection (*n* = 64)	IGRA-negative controls (*n* = 26)	Statistics
Females, n (%)		14 (58)	31 (52)	7 (27)	*P* = 0.066^*b*^
Age, y, mean (range)		38 (18–77)	37 (18–66)	40 (21–70)	*P* = 0.824^*c*^
Non-Swedish born^*e*^, n (%)		19 (79)	57 (89)	11 (42)	*P* < 0.001^*b*^
TB high endemic country, n (%)		17 (71)	43 (67)	9 (35)	*P* = 0.008^*b*^
Time since immigration to Sweden, non-Swedish born y, mean (range)		7 (0–19)	8 (0–40)	9 (1–17)	*P* = 0.729^*c*^
BCG-vaccination yes/no/unknown		7/1/16	17/2/45	7/1/18	*P* = 0.998^*b*^
IGRA result pos/neg/unknown		16/1/6	64/0/0	0/26/0	
Comorbidities^*m*^, n (%)		8 (33)	18 (28)	7 (27)	*P* = 0.862^*b*^
Immunosuppression^*f*^, n (%)		1 (4)	6 (9)	0 (0)	*P* = 0.220^*b*^
Mtb exposure^*g*^	<6 months (contact)	3	16	6	N/A
	<2 years (recent)	6	23	N/A	N/A
	>2 years (remote)	15	25	N/A	N/A
Biochemistry^*i*^^, *j*^ (median, 95% CI)	CRP (mg/L)	22 (1–94)	4 (1–65)	N/D	*P* < 0.001^*h*^
	ESR (mm/h)	43 (4–112)	16 (1–53)	N/D	*P* < 0.001^*h*^
	WBC (10^9^ /L)	6.8 (4–11.8)	6.1 (2.6–11.2)	N/D	*P* = 0.162^*h*^
	Hb (g/L)	126 (93–155)	140 (99–172)	N/D	*P* < 0.001^*h*^
	Alb (g/L)	34 (26–45)	39 (31–44)	N/D	*P* < 0.001^*h*^
Microbiology, n (%)	Sputum microscopy +	7 (58)^*k*^	0	0	N/A
	Sputum Mtb PCR +	8 (67)^*k*^	0	0	N/A
	Mtb culture +	23 (96)^*l*^	0	0	N/A

^*a*^ CRP, C-reactive protein; ESR, erythrocyte sedimentation rate; WBC, white blood cell count; Hb, hemoglobin; Alb, albumin; N/A, not applicable; N/D, not done.

^*b*^ Chi-square test.

^*c*^ One-way ANOVA (Welch’s) test.

^*d*^ TB disease: pulmonary TB n = 13, extrapulmonary TB n = 11 (lymph node, abdominal, and musculoskeletal TB).

^*e*^ Patient origins: *TB disease*: Afghanistan, Azerbajdzjan, China, Eritrea (4), Indonesia, Iraq (2), Mongolia (2), Morocco, Peru, Philippines, Somalia (5), Thailand, Uganda. *TB Infection*: Afghanistan (9), Azerbajdzjan, Chile, DRC (2), Egypt, Eritrea (7), Ethiopia, Gambia (2), Georgia (2), Ghana, India (4), Iran (3), Kirgizistan, Mongolia (4), Pakistan, Peru, Philippines (2), Poland (2), Romania, Russia (2), Somalia (2), Sudan (3), Turkey, Uganda (2), Uzbekistan. Controls: Afghanistan (2), Brazil, Egypt, Eritrea, Ethiopia, India (2), Nepal, Pakistan, Somalia (2).

^*f*^ Immunosuppression: due to medical condition or medical treatments. *TB disease*: chronic kidney disease grade 3. Prednisolone treatment. *TB infection*: methotrexate treatment (5). Chronic kidney disease grade 3.

^*g*^ Exposure: Self-reported or based on epidemiological information, time of arrival in Sweden used as a proxy if time of exposure unknown. Contacts with exposure <6 months identified in contact tracing, household, or other close contacts to a pulmonary TB index.* IGRA-negative controls include* three non-household (school) contacts and three household contacts to low infectious pulmonary TB (PCR + induced sputum = 1 and *Mtb* culture+ in BAL = 2).

^*h*^ Mann-Whitney test.

^*i*^ TBD: ESR missing in 3/24. Alb missing in 5/24. TBI: information complete for 35/64 individuals.

^*j*^ Normal range; CRP <5, ESR <20, WBC 3.5–8.8, Hb >120 (F), >130 (M), Alb 36–48.

^*k*^ In pulmonary TB. In TBI = 13 individuals with negative sputum for Mtb microscopy, PCR, and culture. IGRA-negative controls= 5 individuals with negative Mtb microscopy, PCR, and culture (4 sputum and 1 sputum + bronchoscopy).

^*l*^ Culture negative TBD: 1 PCR+ abdominal TB.

^*m*^ Comorbidities;* TB disease:* Diabetes Mellitus (3), hypertension (3), chronic kidney disease, congestive heart failure, asthma, hypothyroidism, thyroiditis, paroxysmal atrial fibrillation. *TB Infection*: diabetes mellitus (5), hypertension (5), chronic hepatitis B, psoriasis (5), rheumatoid arthritis (2). Chronic kidney disease.

### Comparable detection of Mtb-specific IFNγ-secreting cells with ELISpot and multiplex FluoroSpot

**Fig 1:**
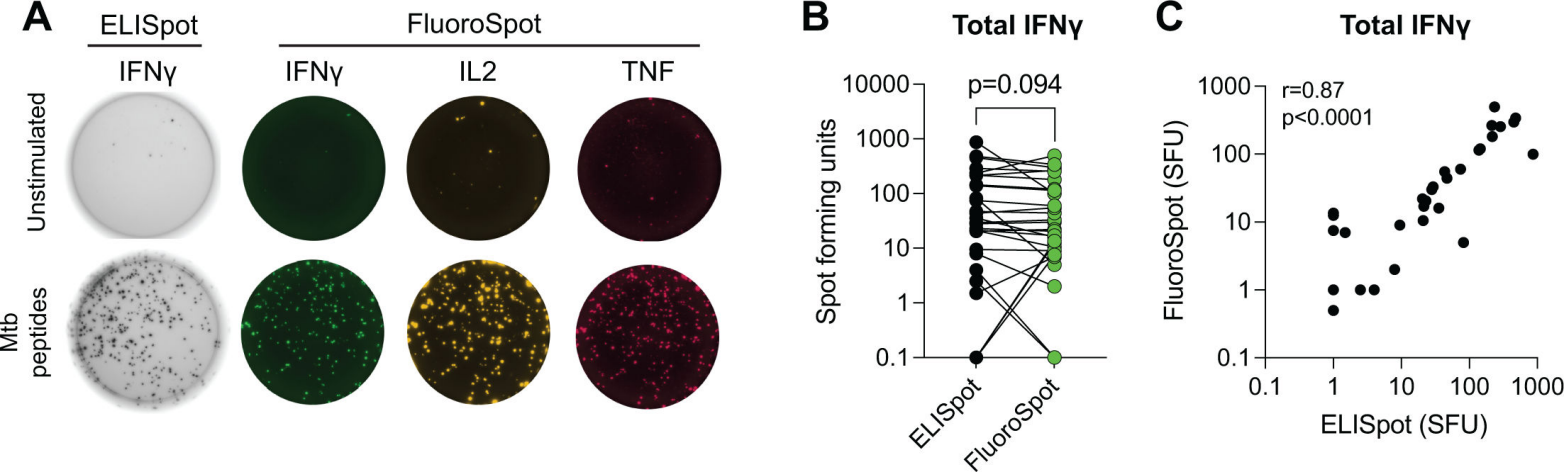
IFNγ, ELISpot, and FluoroSpot results show high agreement in spot-forming units upon stimulation with ESAT-6, CFP-10, and EspC. (**A**) Representative wells from FluoroSpot and ELISpot without stimulation (top row) and with overnight stimulation (bottom row) with peptide pools from Mtb proteins ESAT-6, CFP-10, and EspC. In the bottom row, each colored spot equals one SFU for the respective cytokine measured (IL-2, TNF, and IFNγ). (**B**) IFNγ-specific SFU from paired samples analyzed by both ELISpot and multiplex FluoroSpot. If no spots were detected, values were set to 0.1. Statistical evaluation was done using a Wilcoxon matched pair test. (**C**) Spearman correlation (r) was calculated for IFNγ SFU between FluoroSpot and ELISpot after stimulation. Each dot represents one donor (n = 31). Abbreviations: spot-forming unit (SFU)

The transition from an ELISpot system (used in the T-Spot.*TB* IGRA assay) to a multiplex fluorescent detection of cytokine secretion could potentially impact the detection sensitivity for cytokine secretion. Therefore, we first measured the number of IFNγ-secreting cells, presented as spot-forming units (SFU), from a subset of donors with TBD (*n* = 10), TBI (*n* = 14), or IGRA-negative controls (*n* = 7) using both IFNγ/IL-2/TNF multiplex FluoroSpot and IFNγ ELISpot. PBMCs were stimulated overnight with peptide pools targeting the immunodominant *Mtb*-specific proteins ESAT-6, CFP-10, and EspC, targeted toward MHC class II presentation and memory CD4 T cell activation (Fig. 1A). Comparing the IFNγ detection only, both methods detected IFNγ-producing cells to a comparable extent (Fig. 1B), with the number of SFUs being highly correlated (r = 0.87, *P* < 0.001) (Fig. 1C).

### Lower background when using polyfunctional cytokine secretion to detect Mtb infection

**Fig 2:**
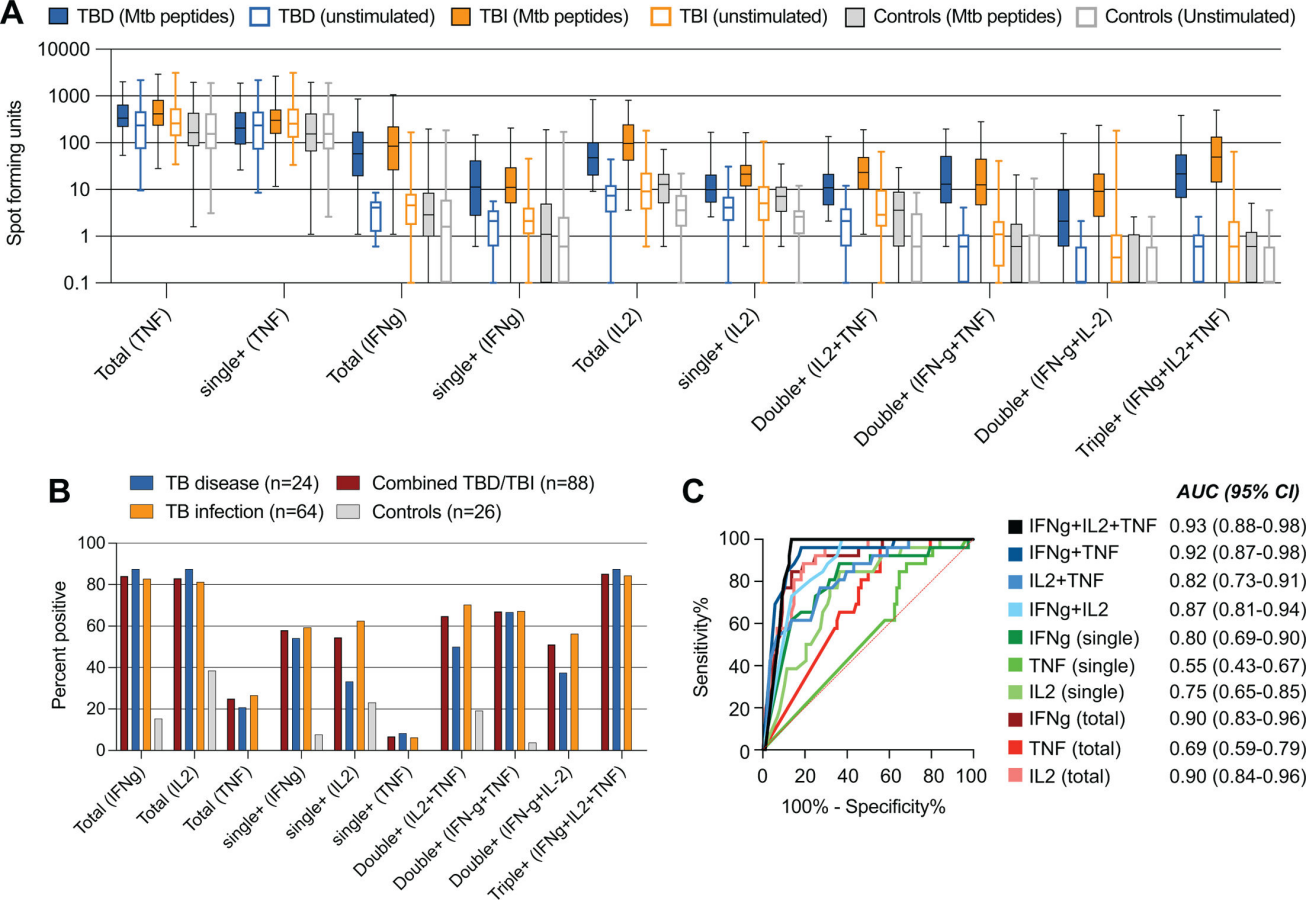
Lower false-positive rates when using IFNγ/IL-2/TNF triple-positive cells for detection of TB infection and disease. (**A**) The number of SFU in ESAT-6, CFP-10, and EspC stimulated and unstimulated conditions for each group (TBD: *n* = 24, TBI: *n* = 64, IGRA-negative controls *n* = 26) and IFNγ, IL-2, and TNF cytokine combination. For donors with no detectable cytokines, the SFU was set to 0.1 to enable visualization on a log scale. (**B**) Frequency of positive donors for individuals with TBD (*n* = 24), TBI (*n* = 64), merged TBD and TBI (*n* = 88), and IGRA-negative controls (*n* = 26). The cut-off for positivity was set as [SFU(stimulated) – 2 x SFU(unstimulated)] ≥5. Total indicates all cells producing the given cytokine, independent of being single, double, or triple producers. (**C**) ROC analysis with calculation of the AUC ±95% confidence interval (CI). TBD and TBI groups combined (*n* = 88) compared to IGRA-negative controls (*n* = 27). Abbreviations: TBD: TB disease; TBI: TB infection; SFU: spot-forming unit; ROC: receiver operating characteristic; AUC: area under the curve; IGRA: interferon gamma release assay.

We next assessed the secretion of the different cytokine combinations with and without stimulation with *Mtb* antigens. *Mtb*-peptide stimulation led to an increased number of SFU for all IFNγ and IL-2 cytokine combinations in both TBD and TBI groups (Fig. 2A). Single-positive IL-2 SFUs were also detectable for IGRA-negative controls, indicating some non-specific IL-2 secretion. TNF was the cytokine secreted by the largest number of cells (Fig. 2A); however, the secretion was equally high in both *Mtb*-stimulated and unstimulated wells and largely came from TNF single-secreting cells. This suggests that TNF single-secreting cells are not *Mtb*-specific, but rather a result of bystander cell activation. To account for this non-specific cytokine secretion, all subsequent calculations of SFUs were background-subtracted with the SFU from the unstimulated wells.

We calculated the percentage of donors that were positive in the multiplex FluoroSpot assay after stimulation with the *Mtb*-peptides (Fig. 2B). To minimize the potential effect of non-specific production of cytokines, we subtracted the background *x2* and defined the criteria for positivity as: *(SFU[stimulated] – 2 × SFU[unstimulated])* ≥5. We chose 5 SFUs as the cut-off because of the T-Spot.*TB* IGRA uses <5 SFU as a cut-off to identify negative wells. Triple-positive IFNγ/IL-2/TNF cells (i.e., all three cytokines detected in the same SFU), total IFNγ, and total IL-2 (i.e., any SFU containing IFNγ or IL-2, respectively, alone or in combination with any other cytokine) yielded the highest frequency in both the TBD group (88%), in the TBI group (81%–84%), and the two TB groups combined (83%–85%) (Fig. 2B). In the IGRA-negative control group, no triple IFNγ/IL-2/TNF-secreting cells were detected, compared to total IFNγ where the criteria for positivity were reached in 4 donors (15%) and total IL-2 in 10 donors (39%), indicating a significantly higher specificity for the triple IFNγ/IL-2/TNF-secreting cells (*P* = 0.0128 by Fisher’s exact test comparing false positivity among IGRA-negative controls).

For double cytokine-secreting cells, overall lower positivity rates were seen in both TBD and TBI compared to triple IFNγ/IL-2/TNF + and total IFNγ and IL-2 (Fig. 2B). Relatively more individuals with TBI were positive for IL-2 combinations compared with individuals with TBD (IFNγ/IL-2: 56.3% vs 37.5%, and IL-2/TNF: 70.3% vs 50%), which was also observed for single IL-2+ cells (62.5% vs 33.3%). Single TNF + stood out by demonstrating low positivity rates in either group (6.3% vs 8.3%). This was primarily due to high background levels indicating non-specific TNF release.

To measure the overall performance of the different cytokine combinations for detecting an *Mtb*-specific immune response, we combined the TBD and TBI groups and compared them to the IGRA-negative controls through an area under curve receiver operating characteristic (AUROC) analysis (Fig. 2C). Based on the number of background-subtracted SFU, AUC was highest for triple IFNγ/IL-2/TNF+ cells at 0.93 (95% CI 0.88–0.98), mainly explained by its higher specificity, followed by IFNγ/TNF (AUC = 0.92, 95% CI: 0.87–0.98), total IFNγ (AUC = 0.90, 95% CI: 0.83–0.96), and total IL-2 (AUC = 0.90, 95% CI: 0.840–0.964) (Fig. 2C).

### Increased classification of Mtb infection using IFNγ/IL-2/TNF triple-secreting cells compared with total IFNγ

**Fig 3:**
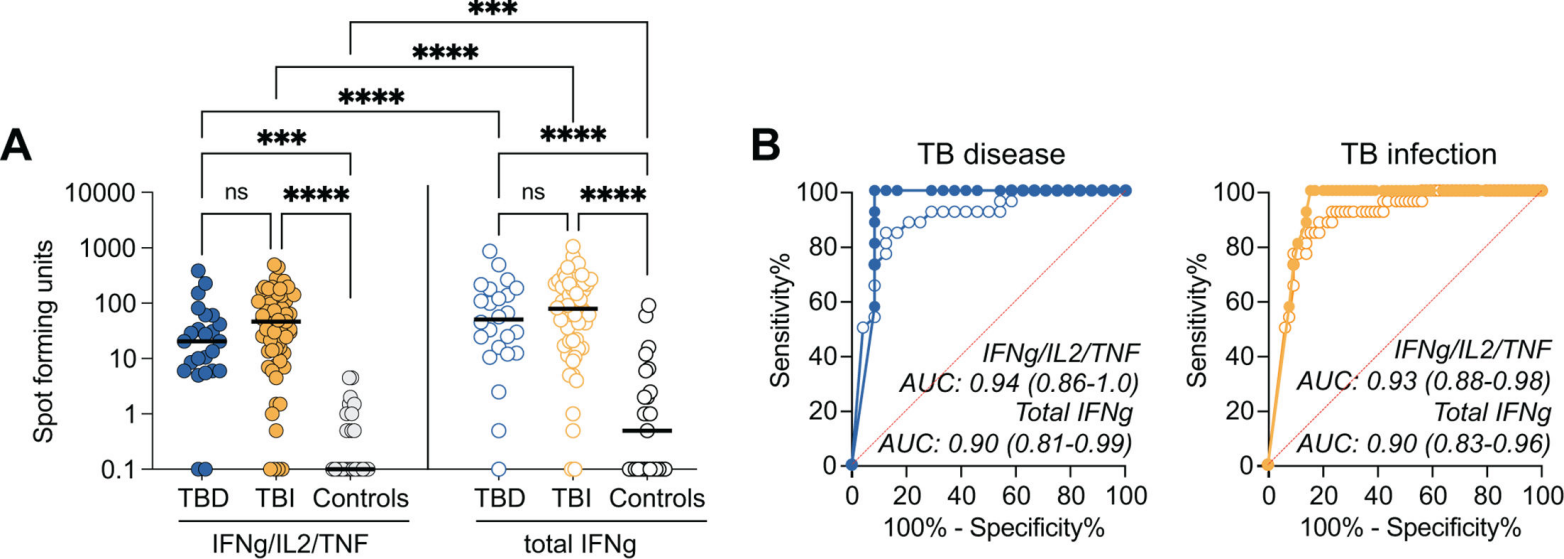
Evaluation of diagnostic performance based on detection of total IFNγ or IFNγ/IL-2/TNF SFUs. (**A**) Background-subtracted SFU for TBD (*n* = 24), TBI (*n* = 64), and IGRA-negative controls (*n* = 26). The left panel indicates the SFU count for cells simultaneously secreting IFNγ, IL-2, and TNF, and the right panel indicates the SFU counts for all cells producing IFNγ, whether as single cytokine or in combination with other cytokines. Donors with no detectable cytokine-secreting cells were set to 0.1. Statistics between independent groups were calculated with a Kruskal-Wallis test followed by uncorrected Dunn’s posttest, while comparison between IFNγ/IL-2/TNF and total IFNγ was calculated based on the Wilcoxon matched-pairs test, ns = *P* > 0.05, ***P < 0.001, ****P < 0.0001. (**B**) AUROC curves for TBD (left panel) and TBI (right panel) comparing IFNγ/IL-2/TNF+ (Triple; filled circles) and Total IFNγ+ (Total; open circles). AUC and 95% confidence intervals are indicated. Abbreviations: SFU: spot-forming units; TBD: TB disease; TBI: TB infection; IGRA: interferon gamma release assay. AUROC: area under the receiver operating characteristic. AUC: area under the curve.

The discovery that the positivity rate for triple IFNγ/IL-2/TNF-secreting cells was similar to total IFNγ-positive cells in both TB groups (TBD and TBI) but lower in IGRA-negative controls (Fig. 2A) led us to explore the performance of the triple+ cells compared to standard IFNγ. We compared the level of SFUs for triple IFNγ/IL-2/TNF+ and total IFNγ+ in TBD and TBI versus controls. Both TB groups had higher triple IFNγ/IL-2/TNF+ and total IFNγ+ SFUs compared with controls, while no difference was seen between TBD and TBI. As expected, triple IFNγ/IL-2/TNF+ SFU were lower compared with total IFNγ+ SFU in all study groups (Fig. 3A). We next performed separate ROC analysis based on SFU levels in TBD and TBI versus controls, resulting in a higher AUC for the triple IFNγ/IL-2/TNF+ in both TBD (AUC 0.94 vs 0.90) and TBI (AUC 0.93 vs 0.90), although not to a significant extent (*P* = 0.48 for TBD and *P* = 0.46 for TBI) (Fig. 3B).

### Diverse functional profiles of cytokine-secreting cells upon peptide stimulation

**Fig 4:**
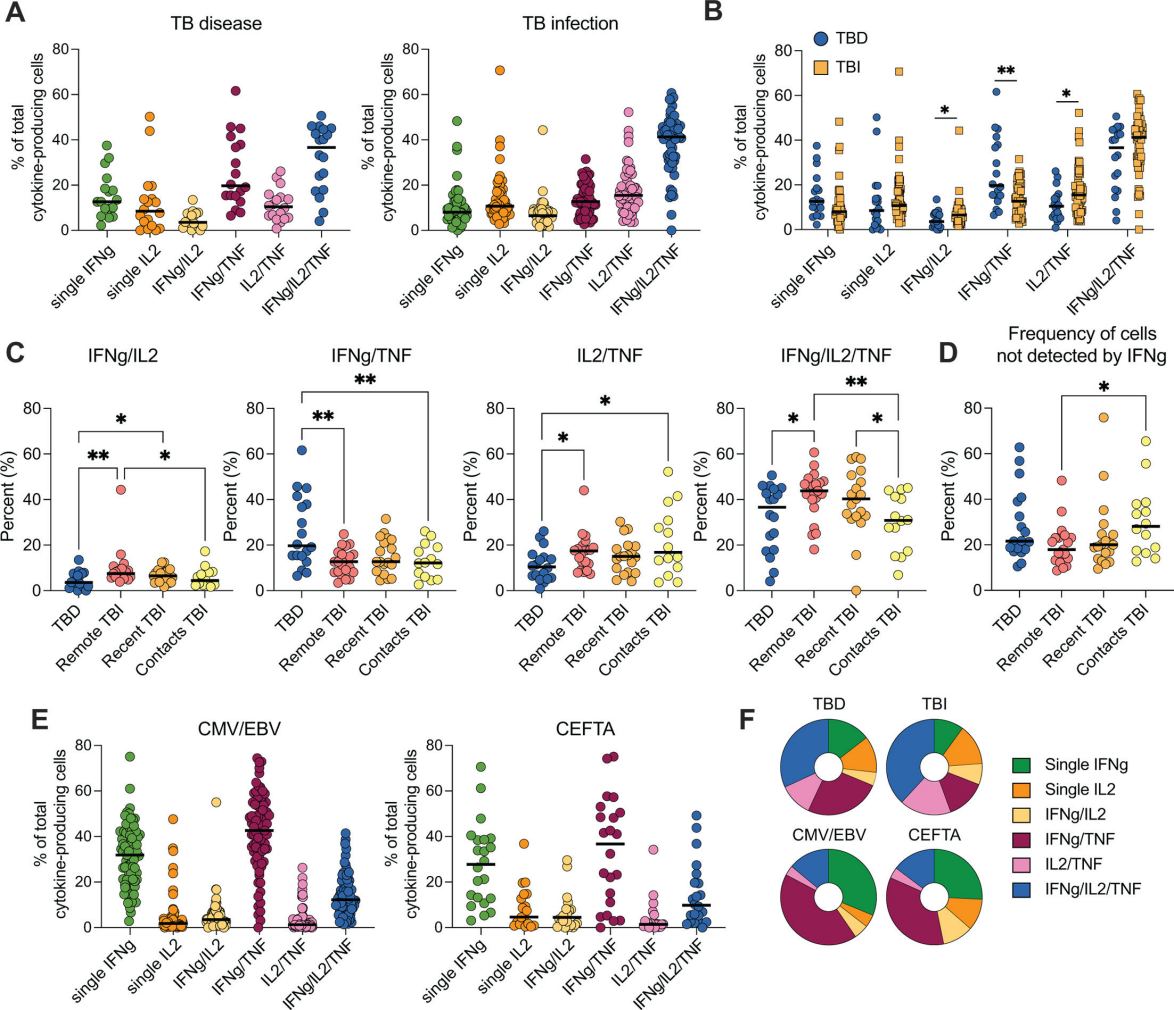
Cytokine combinations in response to pathogen-specific peptide stimulation. (**A**) Frequency of all different cytokine combinations detected after stimulation with ESAT-6, CFP-10, and EspC peptide pools in TBD (left panel) and TBI (right panel). (**B**) The frequency of single, double, and triple cytokine-producing cells after stimulation with ESAT-6, CFP-10, and EspC. Comparison between TBD (*n* = 18) and TBI (*n* = 54). Statistics was evaluated with the Mann-Whitney test. (**C and D**) Frequency of cytokine combinations between TBD (*n* = 18), remote TBI (*n* = 21), recent TBI (*n* = 19), and contacts (*n* = 14). Statistics was evaluated using a Kruskal-Wallis test followed by an uncorrected Dunn’s test. (**E**) Frequency of all different cytokine combinations detected after stimulation with peptide pools targeting T cells specific to CMV and EBV (left panel, *n* = 80) or T cells specific to cytomegalovirus, Epstein-Barr virus, influenza, tetanus, and adenovirus (CEFTA) (right panel, *n* = 22). (**F**) Donut plots indicating the proportion of different cytokine combinations for TBD-, TBI-, CMV/EBV-, and CEFTA-stimulated cells. Donors with <25 total cytokine-producing cells were excluded. *P < 0.05, **P < 0.01. TBD, TB disease; TBI, TB infection; CMV, cytomegalovirus; EBV, Epstein-Barr virus.

To investigate whether the pattern of cytokine secretion differed between TBD and TBI groups, we calculated the frequency of each cytokine combination out of the *Mtb*-specific cytokine-producing cells (Fig. 4). We excluded single and total TNF from the calculation, considering that our results showed it was mainly derived from bystander activation. In both TB groups (TBD and TBI), cells producing all three cytokines were the most common (median TBD: 37% and TBI: 41%), followed by cells co-producing IFNγ/TNF (median TBD: 20% and TBI: 13%) and IL-2/TNF (median TBD: 10% and TBI: 15%) (Fig. 4A). Directly comparing the frequency of cells secreting the different cytokine combinations in individuals with TBD to those with TBI, we found (i) no difference for triple IFNγ/IL-2/TNF+ cells; (ii) more double IFNγ/IL-2+ and IL-2/TNF+ cells in TBI, and (iii) more double IFNγ/TNF+ cells in TBD (Fig. 4B).

We next investigated whether there were differences associated with time since *Mtb* exposure within the TBI group. The group was divided into contacts (<6 months, *n* = 14), recent (6–24 months, *n* = 19), and remote (>24 months, *n* = 21). Within the TBI group, contacts had significantly lower proportions of triple IFNγ/IL-2/TNF + cells compared with both recent and remote TBI, where proportions were gradually increasing (Fig. 4C). The proportion of IFNγ/IL2+ cells was also higher in both recent and remote TBI when compared to TBD, a difference that was not seen when comparing contacts to TBD. Since the IGRA assay is based on IFNγ detection, we also calculated the proportion of cells not secreting IFNγ for each group, with primarily contacts having a larger proportion (median of 28% for contacts) compared with remote TBI (18%) (Fig. 4D).

To understand whether the cytokine profile observed upon stimulation was specific to *Mtb* infection, we investigated the patterns of cytokine secretion when cells were stimulated with peptides from other pathogens. PBMCs from the same donors were stimulated with two additional peptide pools including one for cytomegalovirus (CMV) and Epstein Barr virus (EBV) and one with peptide pools for CMV, EBV, influenza, tetanus, and adenovirus (CEFTA) (Fig. 4E). These mostly virus-specific peptide pools with the addition of peptides for the bacterial tetanus toxoid stimulated primarily production of double IFNγ/TNF+ cells followed by single IFNγ+ cells (Fig. 4E). This contrasted with the *Mtb*-specific responses, which were dominated by polyfunctional IFNγ/IL-2/TNF+ cells and few single IFNγ+ cells (Fig. 4F).

### Adding IL-2/TNF cytokine-producing cells to total IFNγ increases detected SFU levels but does not improve assay accuracy

**Fig 5:**
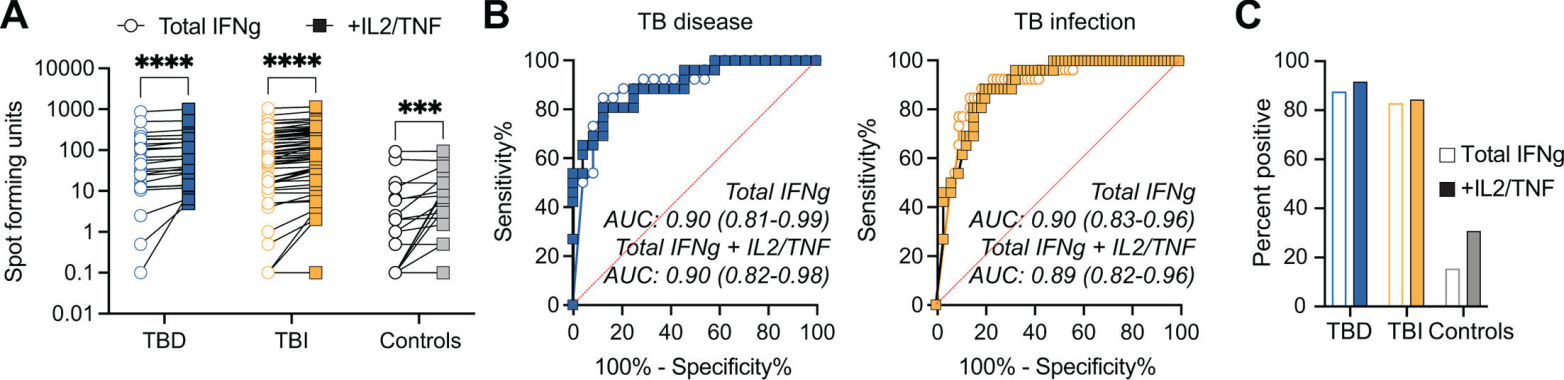
The addition of IL-2/TNF-secreting cells to total IFNγ increases SFUs but not the detection of TB infection or disease. (**A**) Number of (background-subtracted) SFU for total IFNγ (open circles) and total IFNγ with SFUs for TNF/IL-2 added (filled boxes) for TBD (*n* = 24), TBI (*n* = 63), and IGRA-negative controls (*n* = 27). Statistics was evaluated using the Wilcoxon matched pairs test with ***P < 0.001 and ****P < 0.0001. (**B**) AUROC curves for TB disease (left panel) and TB infection (right panel) versus IGRA-negative controls comparing total IFNγ-producing cells (open circles) with IFNγ+ IL-2/TNF (filled boxes). (**C**) Bar graphs indicating detection positivity for individuals with TBD (blue, *n* = 24), TBI (orange, *n* = 63), and IGRA-negative controls (gray, *n* = 27) based on (SFU[stimulated) – 2 × SFU[unstimulated ]≥5) for Total IFNγ alone (open bars) or including IL-2/TNF (filled bars). SFU, spot-forming units; TBD, TB disease; TBI, TB infection; IGRA, interferon gamma release assay; AUROC, area under the receiver operating characteristic.

Since upon stimulation with *Mtb* antigens, on average, 17%–28% of SFUs did not contain IFNγ, we hypothesized that the addition of double IL-2/TNF-producing cells could potentially increase the sensitivity for detecting *Mtb* infection in TB infection and disease. We therefore combined the total IFNγ SFU with double IL-2/TNF + SFU (Fig. 5A). This led to significantly increased SFU in all three groups (TBD, TBI, and controls). We then performed ROC analysis to evaluate whether this would translate to better assay performance in separating TBD and TBI from controls, but AUC remained unchanged in the combined total IFNγ and IL-2/TNF in both comparisons (Fig. 5B). Lastly, we assessed whether additional donors could be identified as assay positive if IL-2/TNF was included in addition to total IFNγ. Indeed, a few additional donors were identified as *Mtb* positive compared to IFNγ alone (TBD: 91.7% vs 87.5%, TBI: 84.4% vs 82.8%); however, the number of false positives also increased greatly (30.8% vs 15.4%), indicating a potential for increased sensitivity at the cost of specificity (Fig. 5C).

### The number of spots and their relative volume correlate with cytokine levels in culture supernatants

**Fig 6:**
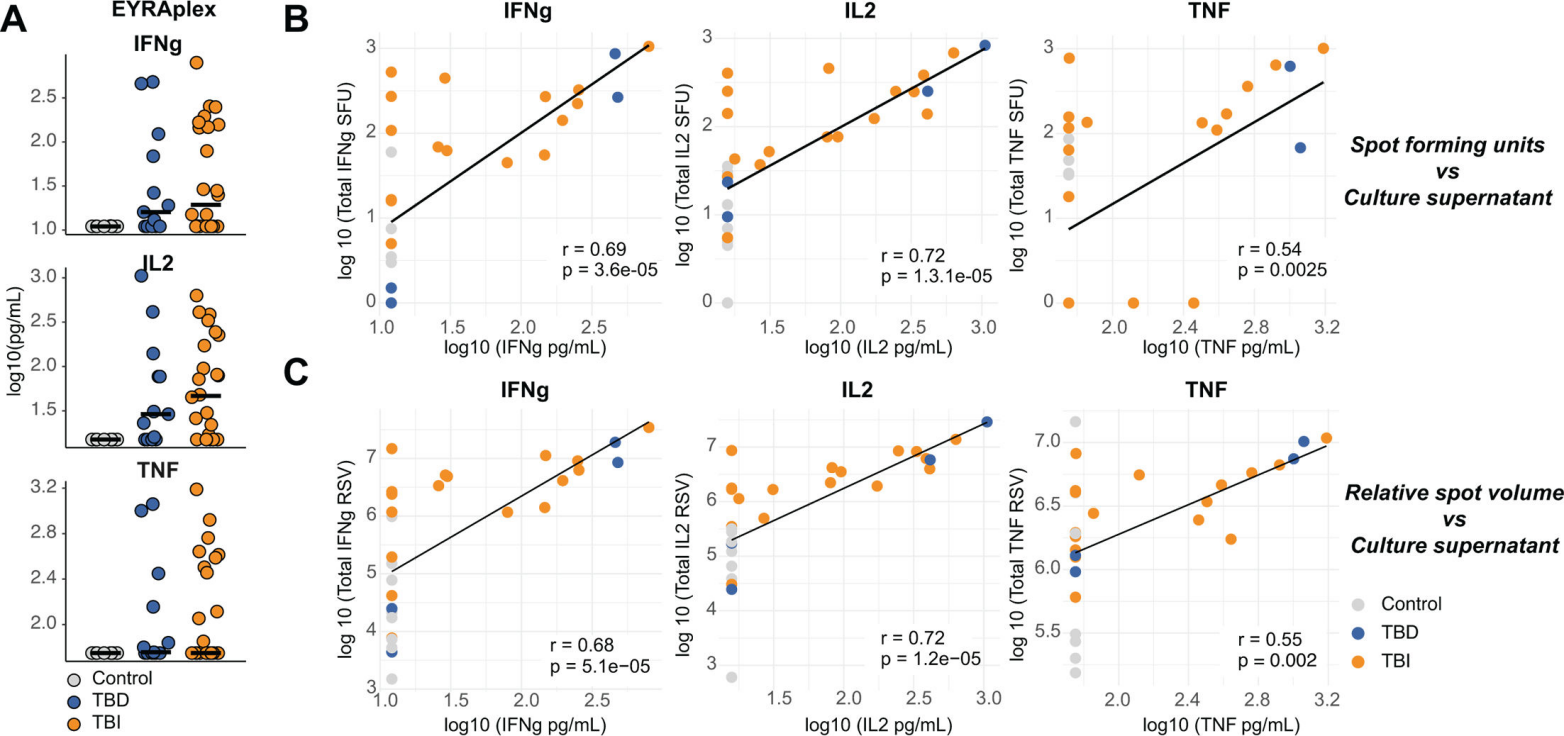
The number of spots and the RSV correlate with cytokine concentrations in culture supernatants after stimulation with Mtb peptides. (**A**) IFNγ, IL-2, and TNF levels were measured in supernatants from IGRA-negative controls (gray, *n* = 9), individuals with TB disease (blue, *n* = 13), and individuals with TB infection (orange, *n* = 24) using EYRAplex after 24 h culture of PBMCs with ESAT-6, CFP-10, and EspC peptide pools. Lines indicate the median. (**B**) SFUs determined by FluoroSpot were log10(y + 1) transformed and correlated with log10-transformed cytokine concentrations from culture supernatants of overlapping donors (*n* = 29). (**C**) Total RSVs were log10-transformed and correlated with log10-transformed cytokine concentrations from culture supernatants of overlapping donors (*n* = 29). Correlation coefficients (R), trend lines, and *P*-values were calculated using linear regression. Abbreviations: SFU: spot-forming units; TBD: TB disease; TBI: TB infection; IGRA: interferon gamma release assay.

In addition to just measuring the number of spots, modern FluoroSpot readers also enable the calculation of spot volume (30). The volume measurement is relative as it does not provide exact concentration measurements per cell, but rather a relative abundance associated with spot size and intensity (36, 37). To ascertain the association between spot volume and secreted cytokine, we stimulated PBMCs from donors of the same cohort (*n* = 46) with ESAT-6, CFP-10, and EspC peptides as done for the FluoroSpot analysis and collected their culture supernatants after 24 h. We measured IFNγ, IL-2, and TNF concentrations using the EYRAplex multiplex bead assay. Overall, FluoroSpot provided increased detection of Mtb-specific responses compared with secreted cytokines, as 24% of individuals with TBD or TBI had cytokine levels below the detection limit at 24 h (Fig. 6A). We then correlated cytokine concentrations with SFUs (Fig. 6B) and relative spot volumes (RSVs; Fig. 6C) for overlapping donors (*n* = 29) and observed significant correlations between the two methods for IFNγ, IL-2, and TNF (Fig. 6B and C). IFNγ and IL-2 both displayed similar high correlations with r-values of 0.68–0.72 (*P* < 0.0001). The correlation for TNF was lower, although also significant (r = 0.53 and 0.55, *P* < 0.01), possibly due to the large number of small spots that might not contribute substantially to cytokine levels in the culture supernatant.

### Quantitative differences in IFNγ, IL-2, and TNF secretion associated with Mtb-infection status and time since Mtb exposure

**Fig 7:**
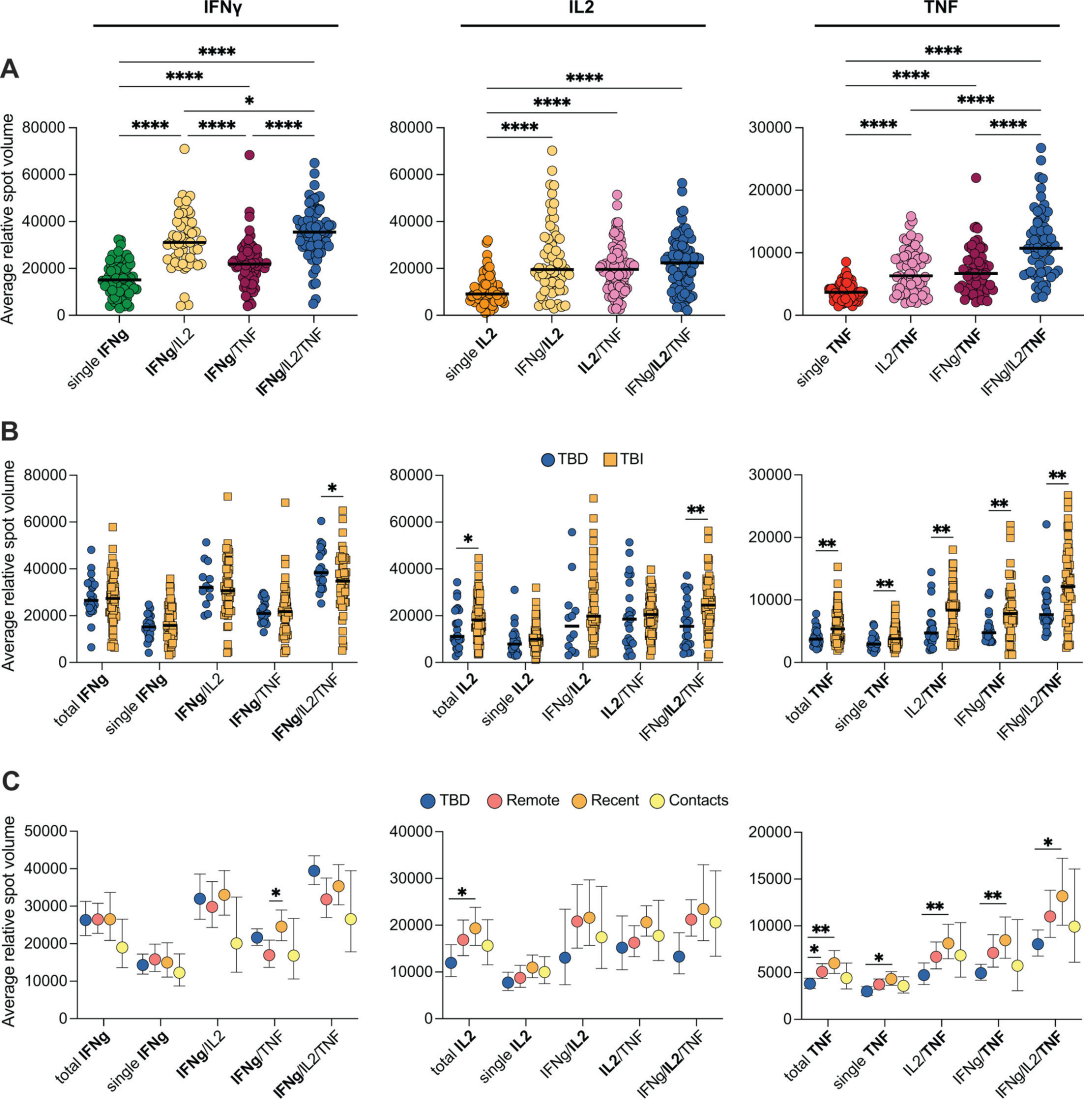
The magnitude of cytokine production is associated with cytokine combinations, Mtb infection status, and time since Mtb exposure. Average RSV for IFNγ (left), IL-2 (middle), and TNF (right) spots in different cytokine combinations for (**A**) TBD and TBI donors combined (*n* = 53–83). Statistics was evaluated using mixed-effects analysis followed by Tukey’s posttest on log10-transformed data. (**B**) TBD (*n* = 12–24) and TBI (*n* = 50–64) donors were separated with statistics evaluated by Mann-Whitney tests. (**C**) TBD (blue, *n* = 12–24) and TBI donors were separated into remote (red, *n* = 22–28), recent (orange, *n* = 16–20), and contacts (yellow, *n* = 12–16) with statistics evaluated using mixed-effects analysis followed by Tukey’s posttest on log10-transformed data. The cut-off to quantify spot volume in an individual sample was to have ≥3 spot-forming units. Lines in A and B indicate the median. Dots represent individual donors. Symbols and error bars in C represent geometric mean ± 95% CI. Only comparisons with *P* < 0.05 are shown. *P < 0.05, **P < 0.01, ***P < 0.001, ****P < 0.0001. Abbreviations: TBD: TB disease; TBI: TB infection.

To better understand how TB infection status might impact cytokine secretion at the cellular level, we assessed the average RSV as a measure of the average amount of cytokine secreted per cell for each analyte and donor. We calculated the average RSV for each cytokine combination in samples with at least three *Mtb* peptide-stimulated spots per well. Overall, in pooled TBD and TBI data, cells secreting only one single cytokine had the lowest spot volume (indicating that the cells produced less cytokine per cell on average), while cells producing all three cytokines had the largest spot volume (indicating on average more cytokine produced per cell) (Fig. 7A). Comparing the dual positive cells, IFNγ/IL-2+ cells secreted more IFNγ compared with cells producing IFNγ/TNF but similar amounts of IL-2 as the IL-2/TNF-positive cells. IL-2/TNF and IFNγ/TNF-positive cells, in turn, produced similar TNF levels (Fig. 7A).

We next separated TBD and TBI and compared the RSV between groups for each cytokine combination (Fig. 7B). Triple IFNγ/IL-2/TNF-producing cells secreted, on average, more IFNγ in individuals with TBD compared with those with TBI. However, the converse was observed for IL-2, where donors with TBI secreted overall more IL-2 and especially among triple IFNγ/IL-2/TNF-producing cells. TNF, on the other hand, was secreted to a larger extent in all cytokine combinations in individuals with TBI (Fig. 7B).

To further investigate whether there were differences in the level of cytokine secretion associated with time since *Mtb* exposure, the TBI group was divided into contacts (<6 months, *n* = 16), recent (6–24 months, *n* = 23), and remote (>24 months, *n* = 25), and spot volumes were compared between TBD and TBI subgroups for all cytokine combinations (Fig. 7C). There was a clear trend for reduced IFNγ secretion for individuals with TBI overall. The effect was more pronounced for polyfunctional cells, specifically, with the lowest levels in contacts. Consistent with the overall group data, individuals with TBD produced less IL-2 compared with TBI. This was observed in all cytokine combinations, although only with statistical support for total IL-2 compared with recent TBI individuals. In contrast, high TNF secretion was primarily associated with recent and remote TBI, while contacts secreted levels similar to individuals with TBD (Fig. 7C).

## DISCUSSION

In this study, we evaluated a FluoroSpot assay with simultaneous detection of the three cytokines IFNγ, TNF, and IL-2 following overnight stimulation with peptides from the *Mtb* antigens ESAT-6, CFP-10, and EspC in individuals with TBD, TBI, and IGRA-negative controls. An inherent challenge when evaluating tests for TBI is that there is no reference method, as current tests are incapable of differentiating between persisting infection and immunological memory after *Mtb* clearance; therefore, sensitivity in TBD can be used as a surrogate measure, and specificity is based on results in unexposed individuals from TB low-endemic settings (15).

Our results indicate that using triple IFNγ/IL-2/TNF+ cells in TBD provided equal sensitivity to total IFNγ detection but had a higher specificity, with no false positives in the IGRA-negative control group. Triple IFNγ/IL-2/TNF+ cells were the most frequently detected in both TB groups (TBD 37% and TBI 41% of SFUs), contrasting with responses to a mixed peptide pool specific to several viral infections, including EBV, CMV, adenovirus, and influenza and the bacterial tetanus vaccine, in which IFNγ/TNF+ cells were the most common. Previous studies have reported partly conflicting findings, with three studies based on flow cytometry suggesting that *Mtb*-specific triple IFNγ/IL-2/TNF+ CD4+ T cells are more frequent in TBD than in TBI and contacts (38–40). Methodological differences and diverse study populations, however, make direct comparisons difficult. Caccamo et al. (39) measured T-cell responses following stimulation with individual *Mtb* antigens (ESAT-6, Ag85B, and 16kD) while we used a combined pool of *Mtb* antigens (ESAT-6, CFP-10, and EspC). Additionally, our TBD cohort included both pulmonary and extrapulmonary TB, while the referred studies all focused on pulmonary TB. Furthermore, one study included only individuals with smear-positive pulmonary TB, with the comparator group consisting of both IGRA-positive and IGRA-negative contacts (38), while another study also included pulmonary TB that had not been culture confirmed (40). In this study, the IFNγ/IL-2/TNF+ cells in TBI also produced less IFNγ compared with individuals with TBD, which could influence identification by flow cytometry depending on how the gates are set.

Somewhat obscuring the interpretation, a negative correlation between triple IFNγ/IL-2/TNF+ cells and bacterial load in pulmonary TB has also been suggested, with levels inversely correlated to sputum smear grade and then gradually increasing during TBD treatment (41) while another study (39) concluded that triple+ frequencies were the same as in controls following successful TBD treatment. One possible explanation could be that when infection progresses from a controlled state (TBI) to a non-controlled state (TBD), the number of triple+ cells peaks, before reducing in numbers and function due to immune exhaustion (42–45), resulting in loss of control of the disease and subsequent high bacterial load. As the pressure on the immune system reduces during treatment with *Mtb,* antigen levels decrease and immune function may be restored, including a contraction of the triple+ T-cell numbers. If that hypothesis is valid, it would mean that reduced numbers of triple+ cells could indicate complete clearance of *Mtb*. If so, this would be useful in the differentiation of IGRA positivity with or without persistent *Mtb* bacilli. Further prospective studies investigating polyfunctional *Mtb*-specific T-cell responses after treatment completion would be important to address this question.

In addition to changes in the levels of cytokine-producing cells, there were also changes in the distribution of cytokine combinations. This could be associated with the *Mtb*-specific CD4+ helper T cells being at varying stages of differentiation, where central memory T helper cells (T_CM_) produce more IL-2 and effector memory T helper cells (T_EM_) produce more IFNγ (46). Consistent with this, we found that individuals with TBD had an increased proportion of cells producing more IFNγ and less IL-2 compared with individuals with TBI, indicative of a higher proportion of *Mtb*-specific T cells at the effector stage. These results support previous findings suggesting a higher number of single IL-2+ T cells in TBI, while triple IFNγ/IL-2/TNF+ and single TNF+ T cells were more common in individuals with TBD than in exposed contacts (38). This is also consistent with observations (39, 47, 48) where IL-2/TNF+ and single IL-2+ cells were associated with TBI, representing *Mtb*-specific T cells at earlier differentiation stages. Additionally, Casey et al. (48) examined IFNγ and IL-2, using a dual FluoroSpot, and found a higher frequency of single IFNγ in TBD and a higher frequency of single IL-2 and double IFNγ/IL-2 secreting T cells in TBI, consistent with our data. Focusing on the triple+ cells’ relative cytokine production, we found that the *Mtb*-specific response in TBD trended toward more IFNγ secretion, while in TBI, IL-2 and TNF were relatively higher expressed. Additionally, the cytokine spectrum of the triple+ cells could provide clues to separate newly acquired TBI from more remote Mtb infection—secreting more IL-2 and less IFNγ and TNF. Measuring non-IFNγ responses could therefore potentially also enable more timely detection of a fresh *Mtb* infection, as contacts exhibited lower IFNγ responses overall.

IL-2 is secreted from activated helper CD4 T cells and serves several roles in T-cell homeostasis, regulation of the function of effector T cells, and stimulating regulatory T-cell differentiation (43, 49). We propose that in TBI, and in particular recent TBI, IL-2-producing central memory T (T_CM_) cells could be more frequent and play a role in mounting an early, efficient immune response to *Mtb* infection. Conversely, upon losing control of the infection, T cells would differentiate toward effector memory (T_EM_) cells with reduced IL-2 production and increased IFNγ and TNF secretion. In support of this hypothesis, our data showed a higher proportion of IFNγ/TNF cytokine-producing cells in TBD compared to TBI. The data are further supported by findings by Arrigucci and co-workers (50), where they observed more IFNγ and TNF mRNA transcripts in individuals with TBD following *Mtb*-specific stimulation and higher ratios of T_EM_ to T_CM_. For our data, the balance between T_EM_ and T_CM_ cells could potentially explain the higher volume of secreted IFNγ in the triple+ cells in TBD, compared to more IL-2 in the triple+ cells in TBI, further indicating a change in IFNγ-producing cells associated with progression toward and development of TBD, previous studies of IFN-γ levels in QTF-plus and QTF-GIT have demonstrated a correlation between higher levels and the risk of progression to TBD (51, 52), potentially indicating a loss of control and expanded differentiation to effector cells. Consistent with these patterns, another study showed that a dual IFNγ/TNF assay improved specificity (94%) for TBD vs TBI and non-TB compared to a single IFNγ assay without affecting sensitivity (47).

In summary, the FluoroSpot assay showed the highest AUC for TBD when using triple IFNγ/IL-2/TNF+ cells, superior to only measuring IFNγ as in the current standard methods. By including multiple *Mtb*-specific responses (IL-2/TNF together with total IFNγ or IFNγ/IL-2/TNF), we could further increase SFU levels, indicating that more *Mtb*-specific cells were detected. This could possibly have a clinical implication by improving the detection of TBI in immunosuppressed individuals who may have reduced levels of circulating T cells, and where current methods might not reach the threshold for a positive test. In this specific patient group, at higher risk of progression to TBD, the corresponding lower specificity could be acceptable. Our findings showed that almost 20% of the *Mtb*-specific cytokine response consisted of IL-2 alone or in combination with TNF and did not include IFNγ, indicating that sensitivity could potentially increase as a wider range of immune responses to *Mtb* antigens are detected. However, as there were not enough immunosuppressed individuals in our study cohort for separate evaluation, additional studies are needed to explore performance in relevant patient cohorts.

The positive predictive value of current IGRA-negative tests and TST for the development of TBD is very low (at best around 5% [17]). This means that a more targeted approach for screening and treatment of TBI in larger populations is needed. Therefore, a potential benefit of a multiplex cytokine assay is that it would enable the detection of a non-IFNγ or more IL-2-dominated immune response in recent *Mtb* infection, of particular importance during contact tracing. Separate patterns related to recent and remote TBI would help identify individuals at higher risk of progression to TB disease and who therefore would have an increased benefit from TBI preventive treatment. Although FluoroSpot, similar to standard IGRAs, depends on laboratory facilities, we see a possible advantage over the single-IFNγ T-SPOT.*TB* assay. By learning more about the different cytokine expression patterns, this could aid in the development of other, less technically demanding, approaches.

### Limitations

In the assay described here, we cannot identify which cell types or differentiation stages of the cells are producing the cytokines. For diagnostic purposes, this is of marginal relevance; however, this limitation does not allow us to fully understand the biological processes involved. In our cohort, we see a discrepancy between the clinical IGRA results and IFNγ-detection in the TBI group, where our assay displays a lower sensitivity. This is probably due to a less streamlined process for handling samples, although strict laboratory protocols were followed. In contrast to the clinical situation where tests are run the same day, our patient samples were stored in the freezer before the experiments were performed. This is supported by our results when measuring cytokines in the supernatant, where detection of *Mtb* responses was further reduced compared with the FluoroSpot, consistent with spot-based methods being more sensitive compared with ELISA or bead-based assays measuring soluble cytokines. Furthermore, the detection of positive study participants was also consistent with other studies done using thawed cells (53). Additionally, even when comparing the different commercial IGRAs head-to-head, the results are not fully congruent.

## MATERIALS AND METHODS

### Study design

Prospective observational cohort study.

### Study participants

Adults (>18 years old) with TB disease or TB infection were prospectively recruited at convenience at the Infectious Disease Department, Karolinska University Hospital, Stockholm, between May 2018 and November 2023. Eligible criteria for the study were as follows: (1) Patients with presumed or confirmed TB disease sampled within 1 week of anti-TB treatment initiation. TB disease was microbiologically verified via *Mtb* culture or *Mtb* PCR. (2) Individuals with TB infection, defined as a positive IGRA result (QuantiFERON-TB Gold In-Tube [QFT-GIT] or QuantiFERON-TB Gold Plus [QFT-Plus]), mainly fall into three categories: (i) close contacts of a TB index case; (ii) screening of migrants from a TB high-endemic country (REF FoHM); and (iii) screening of patients in anticipation of immunosuppression or during pregnancy. TB disease was excluded by chest X-ray, medical examination, and, if warranted, mycobacterial sampling (performed in 13 individuals). (3) IGRA-negative controls consisted of medical students and staff without known or epidemiological risk of TB exposure (*n* = 8), individuals tested via contact tracing (*n* = 6), and patients investigated for TB disease (*n* = 3) or infection (*n* = 10) who upon follow-up had negative IGRA results and TB disease excluded as described for TBI (mycobacterial sampling performed in five individuals).

### Data collection

Demographic, epidemiological, and clinical data for patients with TB disease and TB infection were extracted from patient charts. For all subjects, this included information regarding previous history of or contact with individuals with TB disease, co-morbidities, current medication, and radiological, biochemical, and mycobacterial test results. In TB disease, the presence of typical TB-related symptoms (fever, night sweats, loss of appetite, and weight loss) was noted, and patients were classified as pulmonary or extrapulmonary TB. Microbiological samples for mycobacterial analysis were collected independently of the study in accordance with clinical practice. All patient samples were analyzed at Karolinska University Hospital Laboratory with smear fluorescence microscopy (BX41, Olympus) for detection of acid-fast bacilli, PCR for detection of *Mtb* complex (BD MAX Systems or GeneXpert *MTB* Ultra), as well as mycobacterial culture in liquid (BACTEC MGIT 960) and solid (Löwenstein-Jensen) medium (in-house). Positive microscopy results were graded as high/medium/low per standard laboratory protocol (54).

### Sample collection and processing

Venous blood samples were collected in EDTA vacutainer tubes (BD) and transferred to the laboratory for isolation of PBMCs by density gradient centrifugation, using either SepMate tubes (Stemcell technologies) or Leucosep tubes (Thermo Fisher Scientific). The blood was transferred into 50 mL Falcon tubes and diluted 1:1 with phosphate-buffered saline (PBS) supplemented with 2% heat-inactivated fetal bovine serum (FBS) (Thermo Fisher Scientific). For the isolation with SepMate tubes, 15 mL Ficoll–Paque Plus (Cytiva) was added to 50 mL SepMate tubes and centrifuged for 1 minute at 1,000 × *g* at room temperature (RT). The diluted blood was poured into the SepMate tubes, above the insert, and centrifuged for 10 minutes at 1,200 × *g* RT, with the break on. The cells were then transferred to new 50 mL Falcon tubes. For the Leucosep tubes, 15 mL Ficoll-Plaque Plus was poured into 50 mL Leucosep tubes and centrifuged for 1 minute at 1,000 × *g* in RT. The diluted blood was poured into the Leucosep tube and subsequently centrifuged for 15 minutes at 800 × *g* in RT, with the break turned off. The cell layer was collected using a Pasteur pipette and moved to a new 50 mL Falcon tube. For both methods, the cells were washed twice with 50 mL of PBS + 2% FBS (10 minutes centrifugation at 300 *× g* in RT, brake at 5 out of 9) after collection. After the second wash, the cells were stained with trypan blue and counted using a Countess II automatic cell counter (Invitrogen). The cells were centrifuged and resuspended at a concentration of 5 million cells/mL in freezing medium (FBS + 10% dimethyl sulfoxide) and moved to cryotubes. The tubes were placed inside a CoolCell and stored at –80°C overnight. The following day, the cells were transferred to liquid nitrogen for long-term storage.

### ELISpot and multiplex FluoroSpot

PBMCs were thawed in a 37°C water bath, transferred to 15 mL Falcon tubes, and washed twice (300 × *g,* 5 min) in 15 mL complete media consisting of RPMI-1640 supplemented with 10% fetal calf serum (FCS), 0.1 mM HEPES, 1% penicillin-streptomycin, and 1% L-glutamine (all from ThermoFisher). The cells were left to rest overnight in a 37°C humidified incubator supplemented with 5% CO_2_. On the second day, the cells were filtered through a 70 μm nylon strainer, counted, and resuspended to 5 × 10^6^ cells per mL in complete media.

Pre-coated 96-well FluoroSpot Plus (IFNγ/IL-2/TNF; Mabtech) or ELISpot Plus (IFNγ; Mabtech) plates were washed 5 times with 200 μL PBS (Merck) and blocked with 200 μL complete media for 30 min to 1 h at 37°C. PBMCs were then seeded in 96-well plates at 50 μL per well, corresponding to 2.5 × 10⁵ cells. An additional 50 μL of stimulation was added to each well, giving a total volume of 100 μL per well. After the addition of the stimulation, the plates were incubated overnight at 37°C, 5% CO_2_.

The stimulations consisted of (i) a mixed *Mtb* peptide pool containing peptides from EspC (#3623-1, *n*=23 peptides), CFP-10 (#3625-1, *n* = 23 peptides), and ESAT-6 (#3624-1, *n* = 21 peptides), (ii) a mixed EBV (#3641-1, *n* = 100 peptides) and CMV peptide pool (#3619-1, *n* = 42 peptides), targeting both CD4+ and CD8+ T cells, (iii) a CEFTA peptide pool (#3617-1) consisting of 35 MHC class II-restricted peptides from human CMV, EBV, influenza virus, tetanus toxin, and adenovirus 5, targeting CD4 + T cells designed to stimulate with a broad array of HLA types. All peptide pools were purchased from Mabtech. The peptide stimulations received co-stimulation with 0.1 μg/mL anti-CD28 antibody (Mabtech). Unstimulated cells were used as a negative control. The final concentration of each peptide in the culture was 2 µg/mL.

After 16–24 hours in a humidified incubator with 5% CO₂, the plates were washed five times with 200 μL PBS, and 100 μL detection antibodies (either anti-IFNγ-biotin for ELISpot or a mix of anti-IFNγ-BAM, anti-IL-2-biotin, and anti-TNF-WASP for FluoroSpot) were diluted in PBS + 0.1% BSA and added and incubated for 2 h at RT. After washing the plates five times, 100 μL fluorophore-conjugates (SA-ALP for ELISpot or anti-BAM-490, SA-550, and anti-WASP-640 for FluoroSpot) were diluted in PBS + 0.1% BSA and added for 1 h at RT. After additional washing five times, 50 μL BCIP/NBT-plus was added, followed by 5–10 min incubation before washing in tap water (for ELISpot) or 50 μL fluorescence enhancer was added for 15 min and then flicked out (for FluoroSpot) before plates were dried overnight at RT in the dark. Spot analysis was done on a Mabtech IRIS 2 FluoroSpot/ELISpot reader. The IRIS reader software uses the RAW spot algorithm (30) that, in addition to measuring spot-forming units and size, calculates the RSV of each cytokine based on the three-dimensional spot volume of the different analytes present in a single spot (36, 37). The software allows the user to export average spot volume information from spots with different characteristics, such as single-positive, double-positive, and triple-positive specifically, or from all spots together.

### EYRAplex analysis of cytokines in culture supernatants

PBMCs were thawed and stimulated with the mixed *Mtb* peptide pool or the CMV peptide pool, as described above, except for the stimulation being done in a 200 µL total volume. Briefly, the cells were stimulated with 2 μg/mL of each peptide in a 96-well round-bottom tissue-culture plate (Techno Plastic Products) on the same day as thawing. After 24 hours in a humidified incubator with 5% CO₂, the plate was centrifuged (300 × *g* for 2 min), and the supernatants were collected and stored at –80°C for later analysis. Cytokines in the supernatants were measured using a 22-plex EYRAplex assay (Mabtech). Pre-mixed mAb-conjugated beads were added to the plate (50 µL/well) and washed four times with 200 µL wash buffer. Samples were spun down (300 × *g* for 10 min) and diluted in assay diluent. Samples and standards were added to the plate (50 µL/well) and incubated for 2 hours at RT on an orbital shaker (800 rpm). The plate was washed as described before. Then 50 µL of filtered Detection mAb mix was added to each well before a 1 hour incubation on the orbital shaker as before. The plate was washed again before adding 50 µL of Streptavidin-PE per well and then incubated for 30 min on the orbital shaker. The plate was washed again, followed by the addition of 50 µL of EYRAplex assay diluent, with the plate placed on the orbital shaker for 5 min. Beads were allowed to settle for 20 min before analysis with the Mabtech EYRA instrument. Mabtech Opal software using the RAWsphere algorithm was used to determine each bead ID and translate the respective PE fluorescent signal into analyte concentrations by extrapolating from a standard curve included on the plate.

### Statistical evaluation

Statistical analyses were done using GraphPad Prism version 10.4.2. Overall, due to the nature of the data distribution, SFUs were log10-transformed prior to calculating statistics using parametric tests, or, alternatively, non-parametric tests were used. Data based on proportions or average RSVs were calculated on non-transformed data. Two-way ANOVA or mixed-effects models were used, followed by multiple tests with Tukey’s post-test for parametric data or Kruskal-Wallis, followed by multiple tests with (Mann-Whitney for unpaired and Dunn’s or Wilcoxon for paired) non-parametric data. *P*-values were indicated as *P < 0.05, **P < 0.01, ***P < 0.001, ****P < 0.0001.

## Data Availability

Data is available upon reasonable request to the corresponding author and pending confirmation of relevant permits.
